# Nutritional composition, health benefits and bio-active compounds of chickpea (*Cicer arietinum* L.)

**DOI:** 10.3389/fnut.2023.1218468

**Published:** 2023-09-28

**Authors:** Nabila Begum, Qudrat Ullah Khan, Leyna G. Liu, Wenwen Li, Dahai Liu, Ijaz Ul Haq

**Affiliations:** ^1^School of Medicine, Foshan University, Foshan, Guangdong, China; ^2^Division of Cell, Developmental and Integrative Biology, School of Medicine, South China University of Technology, Guangdong, China; ^3^Greater Bay Area Institute of Precision Medicine (Guangzhou), Fudan University, Guangzhou, Guangdong, China; ^4^College of Letters and Science, University of California, Berkeley, Berkeley, CA, United States; ^5^Reproductive Medicine Center, Department of Obstetrics and Gynecology, The First Affiliated Hospital of Anhui Medical University, Hefei, Anhui, China; ^6^Department of Public Health and Nutrition, The University of Haripur, Haripur, Pakistan

**Keywords:** chickpeas, bioactive compounds, antioxidant, anticancer, *Cicer arietinum*

## Abstract

Chickpea (*Cicer arietinum* L.), an annual plant of the family Fabaceae is mainly grown in semiarid and temperate regions. Among pulses, cultivated worldwide chickpeas are considered an inexpensive and rich source of protein. Chickpea is a good source of protein and carbohydrate, fiber, and important source of essential minerals and vitamins. The quality of protein is better among other pulses. Consumption of chickpeas is related to beneficial health outcomes. Dietary peptides from the protein of chickpeas gaining more attention. Peptides can be obtained through acid, alkali, and enzymatic hydrolysis. Among all these, enzymatic hydrolysis is considered safe. Various enzymes are used for the production of peptides, i.e., flavorzyme, chymotrypsin, pepsin, alcalase, papain, and trypsin either alone or in combinations. Chickpea hydrolysate and peptides have various bioactivity including angiotensin 1-converting enzyme inhibition, digestive diseases, hypocholesterolemic, CVD, antioxidant activity, type 2 diabetes, anti-inflammatory, antimicrobial, and anticarcinogenic activity. This review summarizes the nutritional composition and bioactivity of hydrolysate and peptides obtained from chickpea protein. The literature shows that chickpea peptides and hydrolysate have various functional activities. But due to the limited research and technology, the sequences of peptides are unknown, due to which it is difficult to conduct the mechanism studies that how these peptides interact. Therefore, emphasis must be given to the optimization of the production of chickpea bioactive peptides, *in vivo* studies of chickpea bioactivity, and conducting human study trials to check the bioactivity of these peptides and hydrolysate.

## Graphical abstract

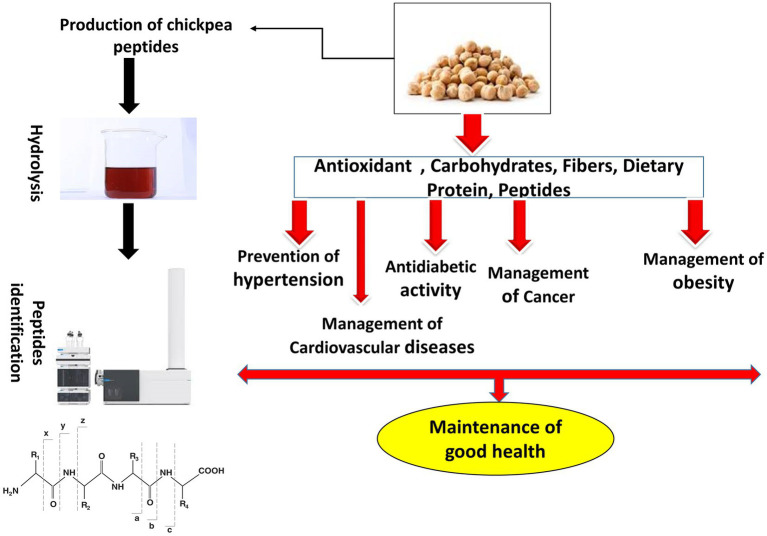

## 1. Introduction

Pulses are also called “meat for poor men,” because of the high content of their protein profile, especially in poor countries of the world, they occupy a unique place. Pulses are leguminous in nature and can be grown in all types of soil, they can help to utilize the surrounding nitrogen in the soil, help to improve the soil fertility, and significantly contribute to economic and environmental sustainability. Chickpeas are considered the most primitive legumes pulse crop cultivated around ~7500 years ago in the Middle East (1).

**Figure 1:**
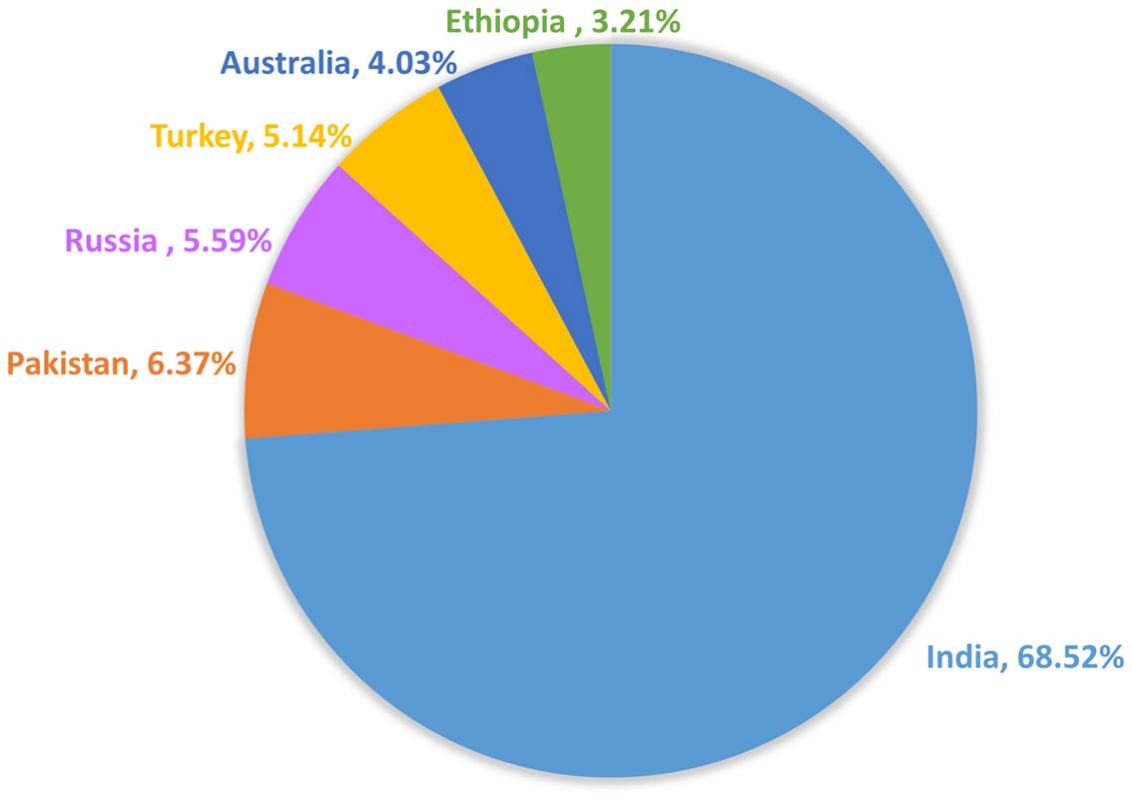
Leading chickpea producers and contributors to the global production.

Chickpea (*Cicer arietinum* L.) which is also called Garbanzo beans is considered a good source of high-quality protein and has good nutritive value for human nutrition. There are mainly two varieties of chickpea (CP) “Desi” and “Kabuli” type. Around 80–85% of the total CP area is covered by the Desi type and the major producing countries are Africa and Asia, while for the Kabuli type the main production countries are Europe, North Africa, West Asia, and North America (2). For the production of chickpeas, India ranked 1st position, with around 6 million tons of production per year, followed by Pakistan. The other chickpea-producing countries are southern Europe, North Africa, Australia, and America. A total of 70% of the world’s chickpeas production comes from Turkey, Iran, Pakistan, and India. Worldwide chickpea is the third most key pulse crop, followed by field peas and dry beans (3). In 2020 the main importer countries for the CP were Pakistan, Bangladesh, and India, whereas Russia, Australia, and Canada were the top exporters (4) (Figure 1).

In developing countries, CP is the most important basket food. It is considered a highly consumed seed food, while the method of preparation depends on regional and ethnic factors. According to the WHO, CP contains a high level of non-starch polysaccharides and a low amount of calories. Lentils, beans, and CP are good sources of minerals, vitamins, and fiber and are recommended as an important part of a healthy diet for all types of populations. CP has good nutritional value and has the potential to be used as a functional food for the management of various diseases (5). Besides, the protein content and the dietary fiber content of the CP are highest among pulses, as demonstrated by various studies fiber rich food consumption is recommended for several diseases (3). Fiber plays an important role in weight reduction and is highly recommended for obese individuals. Moreover, dietary fiber can be helpful to reduce the risk of stroke, type 2 diabetes, coronary heart disease, gastrointestinal diseases, and hypertension (6). Furthermore, previous studies reported the antihypertensive effect of bioactive compounds from CP (7). Proteins obtained from CP are a good and balanced source of essential amino acids, with high bioavailability. Chickpea protein hydrolysate exhibit various biological activities such as reduction of antigenic activity and angiotensin I-converting enzyme (ACE) inhibition (8). Organic compounds such as isoflavones and polyphenols are helpful to reduce the risk of hypertension, inflammation, diabetes mellitus, and metabolic syndrome (1).

Chickpea is one of the major pulse crops and has been used intensively as food in developed countries. Chickpeas have nutritional value for humans and play important role in the management of various diseases. Publication about the nutritional components and bio-active peptide extraction and composition is limited. This review focused on the nutritional composition and bioactive peptide derived from CP, their method of production, and health benefits have been reviewed.

## 2. Composition of chickpeas

Desi and Kabuli are the two distinct varieties of chickpeas. The Desi variety has a wrinkled shape, thick seed coat, and colored flower angular seeds. The seed coat usually contains 14% of the total mass of the grain. The net seed weight for the desi variety ranges from 0.1 g to 0.3 g. the total chickpea production area is covered by the desi variety (80–85%), and the main producing countries are semi-arid areas, i.e., Africa and Asia. The Kabuli variety has a thin seed coat, white flowers, smooth seed surface, seed color is white, and seed weight ranges from 0.2 g to 0.6 g. The seed coat contains 5% of the total seed weight. Kabuli varieties are largely grown in temperate regions, i.e., Europe, North Africa, North America, and West Asia (9).

### 2.1. Chickpea protein content and digestibility

After cereals legumes are the most important food and are considered worldwide as a meat alternative because of their low price and sustainability. Chickpeas contain good-quality protein having higher bioavailability (10). In developing countries of Asia and Africa, protein energy malnutrition in young children and infants is common and can affect their health condition due to lack of energy and protein in the diet. In the Afro-Asian diet, pulses are a major part and can minimize the occurrence of malnutrition by providing a higher protein percentage (11).

Albumins and globulins are the predominant protein found in legumes. The globulin proteins of the legumes contain vicilin (7S), convicilin (15S), and legumin (11S). Other proteins such as prolamines and gluteins are present in small amounts. Prolamines contain high content of glutamine and proline and are soluble in alcohol. Cysteine and methionine concentrations are higher in gluteins, these two are more important amino acids for human health. Gluteins are soluble in reducing salt, base, acids, and detergents (12).

By comparing the protein content of various pulses, the content ranges from 15 to 30%. Among which content for great northern beans were (23–27%), kidney beans had (27%), navy beans and small white beans (26 and 22.7%) respectively (13). While red, pink and pinto beans were (25.3, 22.9, and 21.1%) (14). Cowpeas and black beans protein ranges from 22 to 28% and 22.9–27.7% (15). According to US pulse quality survey peas and lentil protein content were 19–30% (16). The environment and cultivar have a great influence on the overall content of protein in various pulses (17). While comparing the protein composition in edible beans, lentils, pinto beans, great northern beans and kidney beans is globulin (49–80%) followed by albumin (15–25%), glutelins (18–21%), promalins (2–4%) (18). Chickpea protein content varies significantly on dry mass base before (17–22%) and after (25–29%) dehulling. Kabuli and desi type’s protein concentrations show a significant difference in various studies (3). The protein content of the Kabuli chickpea (20.55%) was lower as compared to the desi type (29.2%) (19). While another study carried out by Singh et al. (20) showed that the protein content ranges from 15.7–31.5% (20). Kou et al. (21) revealed that the average protein content in desi and Kabuli was 22.2, and 23.4%, respectively (21). According to (22) the protein content of dried chickpea are (19–27%). Globulins (53–60%) is the major protein in chickpea while other proteins such as albumins (8–12%), promaline, and glutelins are (3–7%) and (18–24%), respectively (22). While another study investigated the protein content of six varieties of dried chickpeas, the content varies from 20.9 to 25.7%. The content of prolamine, glutein, albumin, and globulin were 19.38–24.40%, 3.12–6.89%, 8.39–12.31%, and 53.44–60.29%, respectively (12). As reported by da Silva et al. (23), globulin content in chickpeas was 41.79%, promaline, was 0.48%, gluteins 9.99% and albumin 16.18% (23).

The protein digestibility of the desi type is lower as compared to the Kabuli type. Singh et al. (24), also investigated that protein digestibility of the desi type was lower (63%) as compared to the Kabuli type (79%). According to (25) *in vitro* protein digestibility of raw chickpeas ranged from 34 to 76% (25). Increase *in vitro* protein digestibility was found by Chitra et al. for chickpea (79.4%) as compared to other pulses, i.e., soybean, mung bean, pigeon pea, and red beans (26). Various sources of research showed variability (48–89%) in the protein digestibility of chickpeas (27). The digestibility of chickpea flour can be increased by fermentation. Besides the digestibility fermentation can improve the flavor and textural properties of chickpea flour. The content of essential amino acids including phenylalanine, cysteine, threonine, tyrosine, and methionine was also higher in fermented chickpea flour (28). Geographic location, environmental variability, plant growing season, analysis method by different authors, and variety of chickpeas, all these factors are responsible for the protein composition and amount present in chickpeas (29).

### 2.2. Amino acids composition of chickpea

The nutritional quality of foods can be determined by the amino acids composition. The amino acid composition of great northern, black, kidney, navy, white beans pink and pinto are leucine, cysteine, aspartic acid, glutamic acid, methionine, Lysine and lysine. Navy beans and beans are poor source of histidine, methionine and cysteine (30). Chickpeas contain a high amount of amino acids, but some amino acids are deficient. Few studies show that the limited amino acids are arginine and aspartic acid (31). While other demonstrated that methionine and cysteine are found low in chickpeas (FAO/WHO 1985). However, the deficiency of these amino acids can be overcome to fulfill the dietary requirement of an individual by consuming pulses along with cereals (3). The content of essential amino acids is significantly higher in chickpea flour (39.89 g/100 g of protein) as compared to wheat flour (32.20 g/100 g of protein). Chickpea flour has a higher content of arginine, aspartic acid and glutamic acid, the sum of these three amino acids was 36.85 g/100 g of protein for Kabuli and 34.53 g/100 g of protein for desi variety. Tryptophan and cysteine two essential amino acids were not found in chickpeas in this study (20). The protein content is given in Table 1 while the content of amino acids is presented in Table 2.

**Table 1:** The protein content of the different chickpea varieties.

Sample	Protein content	References
Total of 57 verities of chickpea	17.4–20.5 g/100 g	(7)
Total of 6 verities of chickpea	23.1–25.6%	(1)
Chickpea flour	24.4–25.4%	(12)
16 verities of chickpea	18.71–22.30 mg/100 g	(9)
Desi chickpea	20.3 g/100 g	(32)
Chickpea (Dry)	20.47 g/100 g	(33)
Chickpea (cooked)	8.86 g/100 g	
Chickpea	1.80 mg/100 g of BSAEs	(34)
Chickpea	7.82 g/100 g	Marielle et al. (2018)

**Table 2:** Amino acids content of the chickpeas varieties.

Type of amino acid	Content of amino acid (g/100 g)								
	(12)	(35)		(3)		(1)	(9)	(36)	(22)
E, A.A		Desi	Kabuli	Desi	Kabuli	6 varieties of chickpea (flour)	16 varieties of chickpea	Raw chickpea (g/16 g N)	Raw chickpea (g/16 g N)
Isoleucine	0.36	–	–	3.70	3.90	0.86–0.99	4.04–5.82	4.1	4.1
Leucine	0.48	4.24	2.48	6.30	6.69	0.57–1.81	7.15–8.62	7.0	7.0
Lysine	0.91	7.25	7.63	5.55	5.47	0.72–1.71	5.53–9.92	7.4	7.7
Methionine	0.12	1.41	1.14	2.05	1.92	0.13–0.16	0.27–1.14	1.6	1.6
Phenylalanine	0.42	5.84	4.53	5.42	5.81	1.25–1.45	5.18–9.15	5.9	5.9
Threonine	0.06	4.02	4.02	3.23	3.13	0.53–0.72	3.38–4.44	3.6	3.6
Tryptophan	–	–	–	–	–	–	–	1.1	1.1
Valine	0.38	4.69	3.20	3.60	3.83	0.88–1.04	3.97–7.98	3.6	3.6
Cystine	–	–	–	0.15	0.19	0.14–0.16		1.3	1.33
Tyrosine	0.19	2.87	6.93	2.55	2.63	0.51–0.60	1.87–4.75	3.7	3.7
N.A.A	–	–	–	–	–	–	–		
Alanine	0.26	4.11	3.52	3.40	3.44	0.95–1.14	3.78–5.02	4.4	4.5
Arginine	0.48	8.90	8.84	8.11	8.07	2.22–2.60	–	10.3	10.6
Aspartic acid	0.58	10.73	11.30	10.59	11.66	2–2.95	12.67–28.05	11.4	10.4
Glutamic acid	1.67	14.90	16.71	16.70	20.24	3.7–4.53	14.44–18.95	17.3	17.0
Glycine	0.26	3.90	3.90	3.12	2.54	0.87–1.04	3.66–4.70	4.1	3.4
Histidine	0.24	–	–	2.66	2.00	0.55–0.66	0.78–1.41	3.4	4.1
Proline	0.24	3.63	2.95	3.95	4.04	1.04–1.36	2.41–6.33	4.6	4.9
Serine	0.12	5.40	7.33	4.96	3.39	1.08–1.29	3.36–5.63	4.9	4.6

### 2.3. Carbohydrate and dietary fiber

Pulses contain carbohydrate as a major component about (60–70%) on dry weight bases. In plant-based food dietary fiber is the non-digestible part of the human small intestine. In whole grains, the dietary fiber is carbohydrate (non-digestible) which includes soluble (slowly digested in the colon) and insoluble (metabolically inactive and aids in bowel movement), where it goes under fermentation and helps in the intestinal bacteria growth, no starch polysaccharides such as β-glucans, gums, cellulose, pectin, and hemicelluloses. Other fibers such as lignin, fructans, and oligosaccharides are not recovered by alcohol precipitation (37). Tosh et al. (38) reported that the total content of dietary fiber in chickpea ranged from 18 to 22% of which soluble fiber were 4–8%, and insoluble fiber were 10–18%. The total starch content of chickpeas is also the highest at 60.3% among the legumes with a higher value of 32.3% of amylose. Chickpea oligosaccharides ranged from (5.54–8.82%) in the different cultivars. It included a small amounts of 0.25–0.73% of verbascose, 1.54–3.18% of stachyose, 2.04–5.26% of ciceritol, and 0.42–0.86% of raffinose (38).

Aguilera et al. (15) reported that the total dietary fiber content of chickpeas was the highest (18–22 g/100 g) among the other pulses. In which the insoluble part is about 10–18 g/100 g and the soluble part is 4–8 g/100 g (15). The Kabuli type of chickpea has a lower content of total dietary fiber and insoluble fiber as compared to the Desi type. This could be due to the thinner hulls and seed coat in the Kabuli type only 4·3–4·4% of total seed weight compared to the desi type having thicker hulls and seed coat 11·5% of total seed weight. Other researchers also demonstrated that the content of dietary fiber in chickpeas varies from 18.73–21.86% (39). a-galactoside in various pulses (cowpea, horse gram) include verbascose (11, 15%), raffinose (32, 26%), stachyose (56, 58%). While in great northern beans, pinto beans, kidney beans and navy beans the stachyose content is very low (2.2, 4.2, 3.6, and 2.8%) (40). The content of sucrose in peas, mung beans, great northern beans, pinto, navy, red beans and kidney beans are 1.8 2.6, 3.0, 4.8, 3.1 1.6 and 4.1%, respectively, (41). Content of dietary fiber in different pulses, i.e., lupin, cranberry, mung beans, cow peas and lentil are 39, 15, 5%, 11–24% (42).

Chickpea grains contain a high amounts of mono-di and oligosaccharides. According to Sánchez-Mata et al. (43), the concentration of mono-saccharides in chickpeas were ribose, 0·11 g/100 g, galactose, glucose, 0·7 g/100 g, 0·05 g/100 g and fructose, 0·25 g/100 g. Maltose (0·6%) and sucrose (1–2%) have been reported to be the most abundant free disaccharides in chickpeas. Disaccharides include sucrose 1–2% and maltose 0.6%, while the main oligosaccharides stachyose, ciceritol, raffinose, and a small amount of verbascose. The content of stachyose and Ciceritol in chickpeas were 25% and 36–43%, respectively (43). The content of total dietary fiber and carbohydrates in chickpeas by different researchers are presented in Table 3.

**Table 3:** The content of total dietary fiber and carbohydrates in chickpeas by different researchers.

Compound	Content (g/100 g)					
Monosaccharides	(36)	(44)		(15)	(45)	(36)
		Kabuli	Desi			
Ribose	0.32–0.97	–	–	–	–	–
Fructose	0.03–0.19	–	–	3.1	0.1	–
Glucose	0–0.065	–	–	0.5	0.1	–
Disaccharides						
Sucrose	1.09–2.28	3.10–4.41	1.56–2.85	15.2	4.3	1.89
Maltose	0.16–0.68	–	–	3.3	–	–
Oligosaccharides						
Raffinose	0.62–1.45	0.48–0.73	0.46–0.77	3.2	1.0	1.45
Ciceritol	2.51–2.78	–	–	27.6	–	–
Stachyose	0.74–2.56	1.76–2.72	1.25–1.98	17.7	2.8	2.56
Verbascose	0.019	–	–	–	Trace	0.19

### 2.4. Fat content and fatty acid composition of chickpea

Generally, the content of lipids is lower in pulses, i.e., 1–3% in peas, 2–3% in mung beans, and 2% in cowpea. The TAG and PhL content in kidney beans, peas and mung beans are (34–46%, 31–40, 31%) and (49–59%, 52–61, 31%), respectively (46). However, chickpea presents higher content of lipids content up to 7% (22). In pulses, lipids are usually present in the form of sterols, phospholipids (PhL), triacylglycerol (TAG), steryl esters, and free fatty acids (FFA). The phospholipids (PhL) content of chickpeas was around 17–20% and TAG content was 56–67% as reported by Zia-Ul-Haq et al. (29). The fatty acid profile of chickpeas revealed the content of linoleic and oleic acids (nutritionally essential fatty acids), both are present in high amounts. Linoleic acid content was highest followed by palmitic acid and oleic acid. Two important essential fatty acids required for physiological functions, growth, and maintenance are linolenic acid and Linoleic acid. Linoleic acid regulates smooth muscle contraction, reducing blood pressure through the production of prostaglandins. In chickpea composition majority of the fatty acids are unsaturated while saturated fatty acids contribution is minor. This makes the chickpea an exceptional legume from a nutritional point of view. Summo et al. (7) investigated three varieties of chickpeas. Among these, beige chickpea have the highest content of monounsaturated fatty acids as compared to black and brown chickpea. While the content of polyunsaturated fatty acid was the lowest (50.3%) in the beige variety. The content of polyunsaturated fatty acid was significantly higher in the brown (67.0%) and black (64.4%) varieties. This may be due to the higher concentration of linoleic and linolenic acid in brown and black chickpeas (7).

According to Barbana and Boye (31) the lipid content ranges from 4.5 to 6.0 g/100 g. In neutral lipids, the major component is triglycerides while in polar lipids the major component is lecithin. High levels of essential unsaturated fatty acids linolenic acid (0.5–0.9% in oil), linoleic acid (54.7–56.2% in oil), oleic acid (21.6–22.2% in oil), and low level of stearic acid (1.3–1.7% in oil) and palmitic acid (18.9–20.4% in oil) were observed. Other minor amounts of fatty alcohols, waxes, and sterols are also present in chickpea lipid composition. The lipid composition of chickpea is tabulated in Table 4.

**Table 4:** Lipid/Fatty acid profile of chickpea.

Fatty acid	% in oil						
	(29)	(47)	(44)		USDA (g/100 g)	(22) (%)	(29) Desi variety
			Kabuli	Desi			
Palmitic (C16:0)	18.9–20.4	12.7	9.41	9.09	0.51	14	17.8–21.5
Palmitoleic (16:1)	0.3–0.5	0.1	0.30	0.26	0.012		0.3–0.9
Stearic (C18:0)	1.3–1.7	1.5	1.42	1.16	0.085	2	0.9–1.8
Oleic (C18:1)	21.6–22.2	19.3	32.56	22.31	1.346	25	20.9–24.4
Linoleic (C18:2)	54.7–56.2	62.9	51.20	61.62	2.593	57	52.9–55.2
Linolenic (C18:3)	0.5–0.9	3.3	2.69	3.15	0.101	2	0.3–1.01.0–1.8
Arachidic (C20:0)	1.0–1.4	Trace	0.66	0.51	–	3	–
Oleic acid/linoleic acid	0.39–0.41	–	–	–	–	–	–

### 2.5. Minerals content

High content of magnesium, potassium, manganese and iron are found in pulses. Other minerals include calcium, selenium, zinc and copper. According to (48), field peas have an average of 0.047, 1040, 301, 5.4, 117, and 1.3 (mg/100 g) of selenium, magnesium, manganese potassium, iron and zinc content. Mineral composition of lentil include iron (6–9 mg/100 g), calcium (59–69 mg/100 g) potassium (844–943 mg/100 g) of eight different cultivar (49).

Like other pulses, chickpeas not only provide variation in the daily diet of Asian and African people but also provide essential minerals and vitamins. As an important component of enzymes and cells, minerals involves in the majority of defense mechanisms. They act as activators, inhibitors, and regulators of metabolism. In chickpea, the content of zinc, phosphorous, and manganese is higher as compared to other pulses (10). As a good source of zinc and iron chickpeas can play a vital role to overcome the malnutrition of minerals. The content of iron and zinc in chickpeas ranged from 4.56 mg/100 g to 9.87 mg/100 g and 0.96 mg/100 g to 4.05 mg/100 g (10). The content of Fe and Zn ranged from 4.6 mg/100 g to 6.7 mg/100 g and 3.7 mg/100 g to 7.4 mg/100. Other minerals calcium, magnesium, manganese, and copper content ranged from 40.83 mg/100 g to 260.95 mg/100 g, 71.96 to 187.86 mg/100 g, 1.31 mg/100 g to 3.69 mg/100 g, and 0.13 mg/100 g to 1.07 mg/100 g, respectively. Wild species of chickpea have a higher content of manganese, magnesium, and calcium as compared to genotypes. Magnesium and calcium content ranged from 125 to 159 mg/100 g and 93 to 197 mg/100 g. Manganese and copper content in 28 chickpea genotypes ranging from (1.3 to 2.1 mg/100 g) and (0.66 to 1.04 mg/100 g), respectively. Zia-Ul-Haq et al. (29) investigated the minerals content of 4 desi chickpea varieties grown in different areas of Pakistan. The content of potassium ranged from 1109 mg/100 g to 1236 mg/100 g. All varieties have a notable amounts of copper, calcium, and zinc. According to these results, chickpeas can provide an adequate amount of minerals to fulfill the human body’s mineral requirement.

According to (3) 100 g of raw chickpea seed provides approximately 160 mg/100 g of Ca, 138 mg/100 g of Mg, 4·1 mg/100 g of Zn, and 5·0 mg/100 g of Fe. The daily requirement of zinc, iron, and magnesium can be fulfilled through chickpea 100 g/day consumption (3). Desi and Kabuli chickpeas showed no significant difference and minerals content, only in the case of calcium Kabuli type have a lower concentration as compared to the desi type. The minerals composition of chickpea is presented in Table 5.

**Table 5:** The mineral composition/content of chickpea (mg/100 g).

Minerals	(50)	(39)		(44)		(12)		(35)		(7)	(22)	(29)	(36)	(33) (57)		(58)
		Desi	Kabuli	Desi	Kabuli	Desi	Kabuli	Desi	Kabuli					Raw	Cooked	11 chickpeas verities
Cu	1.18	1.25	1.20	1.00	1.00	–	–	0.58	0.7	0.13–0.47	–	10.7–12.2	1.10	0.65	0.35	–
Fe	4.60	4.51	4.46	5.90	5.50	4.59	5.50	48.26	51.11	5.80–9.87	4.6–7.5	2.4–4.1	7.72	4.31	2.89	5.86–6.68
Zn	6.11	3.57	3.50	3.60	4.40	4.07	3.40	3.32	4.18	1.63–4.05	3.4–4.4	3.5–6.0	4.32	2.76	1.53	2.17–2.57
Mn	1.21	1.72	1.65	3.40	3.90	3.81	3.28	3.71	3.88	1.31–4.07	–	1.2–2.3	2.11	21.3	1.03	–
Ca	220.0	210.0	154.0	161.70	106.60	165.0	81.7	177.94	187.25	53.7–260.95	82–272	185–219	176	57	49	164.31211.67
Mg	119.0	128.0	122.0	169.10	177.80	169.0	147.0	133.63	115.53	71.96–180.06	147–195	114.3–115	176	79	48	106.71–123.90
Na	–	22.9	21.07	–	–	–	–	7.35	11.26	–	–	96–107	121	24	7	–
K	–	878.0	926.0	1215.70	1127.20	994.5	1060.0	–	–	–	994–1264	1109–1272	870	718	291	–
P	398.0	–	–	377.30	505.1	451.5	394.0	–	–	–	394–452	246–263	226	252	168	–

### 2.6. Vitamins content of chickpea

A minute amount of vitamins is required per day, a well-balanced combination of pulses, vegetables, meat, fruits, dairy products, and cereals can fulfill these requirements. Pulses are considered a good source of water-soluble vitamins especially vitamin B. In baked products, the use of pulses improves the baked quality of the food items and also provides a high folate content. The folate content of pulses varies from one type to another, in addition, it is influenced by cultivar, composition, and growing location (51). A notable difference is found among pulses folate content. Not only the pulse type, the type of cultivar and growing location also influence the folate concentration. The content of folate in lentils, field peas, yellow peas and green peas were 146–290 μg/100 g, 26–202 μg/100 g, (41–55 μg/100 g) and (50–202 μg/100 g), respectively (52). While in mung beans, cow peas and kidney beans the content was (141–169 μg/100 g) and 96 μg/100 g and 103 μg/100 g (53). Content of thiamine and riboflavin in lupin and lentil was (360–390 μg/100 g, 560 μg/100 g) and (610–650 μg/100 g, 280 μg/100 g) (54). Other vitamins such as niacin and vitamin C has been observed in very minute amount.

Chickpea is a good source of tocopherol and folic acid, other water-soluble vitamins (riboflavin, pyridoxine, and pantothenic acid). The concentration of these vitamins was observed highest as compared to other pulses. According to (22) the folate content in field peas was 26–202 μg/100 g, and in lentils the content was 146–290 μg/100 g. Various peas classes having variability on folate content, green peas (50–202 μg/100 g) having higher folate content as compared to yellow peas (41–55 μg/100 g) (22). Chickpeas have higher folate content of 42–537 μg/100 g. In pulses, riboflavin and thiamin are also present in high concentration. The content of thiamin in chickpeas was 453 μg/100 g, while riboflavin content was 173 μg/100 g. The content of niacin, riboflavin, pyridoxine, and thiamin was reduced during cooking. Leaching and chemical destruction lead to the loss of vitamins. Loss of vitamins by various cooking methods is different. High content of vitamin loss occurs due to boiling as compared to microwave cooking and autoclaving. Table 6 shows the vitamin content of chickpeas.

**Table 6:** Vitamins in chickpea seeds (μg/100 g).

Vitamins	(36)				(3) (mg/100 g)		(22)	(55)	(56)	(44)		(33)		(59)
	Raw	Boiling	Autoclaving	Microwave cooking	Kabuli	Desi				Kabuli	Desi	Raw	Cooked	
Riboflavin	173.33	84.00	90.33	101.33	0.26	0.21	173	0.15–0.30	–	0.26	0.21	0.21	0.06	60
Thiamin	453.33	153.33	161.00	192.00	0.49	0.29	453	0.028–0.40	–	0.4	0.29	0.47	0.11	26.80
Niacin	1602.67	69.33	82.33	223.33	1.22	1.72	1,602–3,900	1.6–2.90	–	1.22	1.72	1.54	0.52	–
Pyridoxine	466.33	266.33	306.33	375.00	0.38	0.30	–	0.55	–	0.38	0.30	0.58	0.13	90.40
Folate	–	–	–	–	299.0	206.5	42–537	150.0	–	299.21	206.48	557	172	167.62
Vitamin A	–	–	–	–			–	–	–	–	–	67	27	–
Vitamin C	–	–	–	–	1.34	1.65	–	2.15–6.00	–	1.36	1.65	4.0	1.3	–
Vitamin D	–	–	–	–			–	–	115	–	–	0	0	–
Pantothenic acid	–	–	–	–	1.02	1.09	–	–	–	1.02	1.09	1.58	0.28	138.70
Cynocobalamin	–	–	–	–			–	–	–	–	–	0	0	–
Biotin	–	–	–	–			–	–	–	–	–			–
Vitamin K	–	–	–	–			–	120	23.2	–	–	9.0	4.0	–

### 2.7. Phytochemical composition

Flavonoids, tannins, isoflavones and phenolic acids are the main phenolic compounds found in pulses. Polyphenol content of lentil, mung beans and anchor was 49.45, 98.02, and 48.41 of mg/100 g of GAEs. Tannins contents varies 213.97, 447.98, 212.52 mg/100 g of TEs. Flavonol and flavonoid content of lentil, mung beans and anchor were 0.05, 0.04, 0.02 mg/100 g of QEs and 475.07, 734.08, 771.35 mg/100 g of CEs (34).

Chickpeas have a good reservoir of bioactive compounds, and having health protective value (7). Flavonoids and polyphenols are two important phytochemical presents in chickpeas in higher quantities, these two compounds act as good antioxidants. The content of flavonoids and polyphenols depends on the chickpea color, the darker the color, the higher will be the concentration. Method of polyphenols extraction, reagents used in extraction, extraction time, and analysis methods all these factor affect the content of phenolic compounds in chickpea. Polyphenolic compound ranges from 0.72 to 1.81 mg/g, anthocyanin content was 14.9 mg/kg of beans. The content of anthocyanins and polyphenols is lower in chickpeas while phenolic acids such as hydroxycinnamic, anise, caffeic, cinnamic, p-coumaric, chlorogenic isoferulic, and piperonyl are present in high amounts. Strong antioxidant properties (reduce oxidative stress, chelate metal ions) is characterized by phenolic acids present in chickpea (60).

Zhao et al. (1) reported the content of total flavonoids, total phenolics, and anthocyanins in 6 different chickpea varieties (1). Results reveal that with coat pigmentation color intensity the content of flavonoids, total phenolics, and anthocyanins was higher as compared to the light-colored coat. In terms of flavonoids in six species, the content ranged from 0.021 ± 0.002 to 0.1 ± 0.01 mg Rutin/g. Content of carotenoids in black and brown chickpeas showed significantly higher values (36.4 mg/kg and 35.2 mg/kg, respectively) compared to beige type. Other researchers also reported that the content of carotenoid was higher in the desi type as compared to the Kabuli chickpea, due to the intensity of seed coat color (51). Anthocyanin content was higher in the beige type as compared to brown. The content in black chickpeas ranged from 23.3 to 159 mg/kg. In terms of total phenolic compounds interestingly no significant difference was found, in brown chickpeas the content was 0.8 mg/g and in black chickpeas was 0.7 mg/g. The daily intake of bioactive compounds and dietary fiber can be improved by the intake of brown and black chickpeas. Positive health benefits for human health can be obtained as chickpeas have a lower glycemic index, and help to lower the risk of diabetes and heart diseases (25.3 mg/kg) (7). Another study carried out by Kaur et al. (10), reported that major phenolic compounds presents in chickpeas are flavonoids, phenolic acids, and condensed tannins (10). These compounds play an important role in the prevention and elimination of existing ROS (reactive oxygen species) from blood. Phenolic compounds have other health benefits such as anti-inflammatory, anti-thrombotic, immune-modulating, anticarcinogenic, antioxidant, cardioprotective, anti-ulcer, anti-atherogenic, analgesic agents, anti-microbial, and anti-allergenic. The content of flavonol in chickpeas ranged from (0.80 mg/100 g to 24.20 mg/100 g). In the wild type, the content was highest (7.94 mg/100 g to 24.20 mg/100 g), followed by desi genotypes type from (5.04 mg/100 g to 13.18 mg/100 g) and desi type (95.04 mg/100 g to 13.18 mg/100 g). The TPC (total phenol content) in chickpeas in different varieties ranged from (27.48 mg/100 g to 113.30 mg/100 g). The content of TPC was lower in the Kabuli type 27.48 mg/100 g to 48.01 mg/100 g, while in the desi type the content ranged from 38.59 mg/100 g to 83.52 mg/100 g. In the wild type, the content varies from medium to high, ranging from 63.08 mg/100 g to 113.30 mg/100 g. Due to the dark-colored seed coat of wild and desi types the TPC was higher as compared to the Kabuli type. The details of bioactive compounds in chickpea is given in Table 7.

**Table 7:** Bioactive compounds in chickpea.

	(1)	(7)	(10) (mg/100 g)	(61) (μg/g)		(34) (mg/100 g CEs)
	6 varieties of chickpea			Kabuli	Desi	
Anthocyanin (mg/kg cyaniding3-O-glucoside)	40–66	29.6–77.8	–	–	–	22.22
Total phenolic (mg GAE/g extract)	1.23–1.67	0.7–0.8	27.48–113.30	1156	2740	–
Flavonoid (mg Rutin/g extract)	0.021–0.1	–	0.80–20.31	313.90	622.50	232.17
Total carotenoids (mg/kg β- Carotene)	–	25.3–36.4	–	21.36	37.19	27.27

## 3. Bioactive peptides from chickpeas

Traditionally for the growth and maintenance of body functions, dietary protein is required which provides energy as well as a balanced source of essential amino acids. The consumption of different compounds, provides beneficial health effects, these compounds are stated as bioactive compounds (62). Plant and animal sources are considered could be good sources of bioactive peptides. In the parent protein sequence, bioactive peptides are specific protein fragments that are active, after enzyme, and acid/base hydrolysis they are released and have numerous biological functions. The molecular weight of these peptides is <6 kDa and usually contains 2–20 amino acids. The activity of these peptides is highly dependent on the sequence and composition of amino acids, these peptides possess various activities such as antioxidative, mineral binding, antimicrobial, hypocholesterolemia, immunomodulatory, and antihypertensive (63). In the past 20 years, various enzymes such as flavorzyme, pepsin, papain, chymotrypsin, and alcalase are used to produces chickpea bioactive peptides. Chickpea peptides have various activities such as hypocholesterolemic, antifungal, anti-inflammatory, anti-obesity, antioxidant, hypoglycemic, and antihypertensive (64).

### 3.1. Production of bioactive peptides

**Figure 2:**
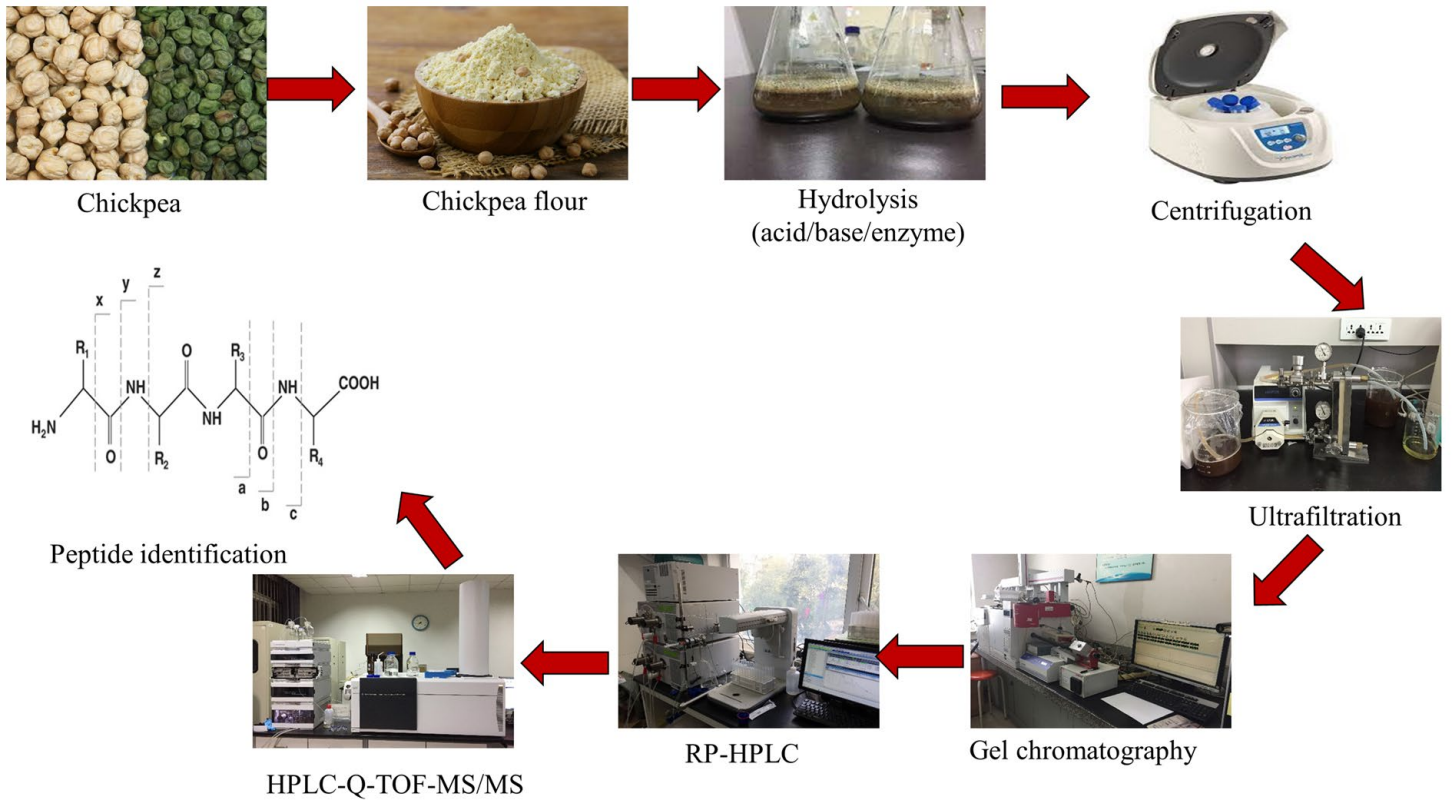
Flow chart for the extraction, purification and identification of peptides.

Chickpea is the second largest producing pulse crop worldwide. It is a good source of essential amino acids and contains a higher percentage of dietary protein 15–30%. For the production of chickpea hydrolysate and peptide, numerous methods have been applied, which improve the functional properties such as angiotensin I-converting enzyme (ACE) inhibitory activity, metal-chelating ability, antihyperlipidemic, antioxidant, antiproliferative, and antitumor activity (65). Flow chart for the peptide extraction, purification and identification is summarized in Figure 2. Through hydrolysis of protein, peptides can be produced, which can be done through acid–base or enzymatic hydrolysis (either by enzymes obtained from plants and microorganisms or digestive enzymes) and microbial fermentation. In South East Asian countries (Japan, Korea, and China) fermentation is the ancient way used for food preservation. Besides increasing the shelf life, fermentation can improve the nutraceutical value of food, this is because microbial proteases generate bioactive peptides from protein fragments (66). Usually, enzymatic hydrolysis is used for the production of peptides, but as compared to acid–base hydrolysis enzyme hydrolysis is costly. Enzymatic hydrolysis improves nutritional properties as well as functional properties such as emulsifying properties of hydrolyzed protein. Chickpea protein concentrate or isolate and chickpea flour can be used for the production of peptides. Chickpea protein isolate and protein concentrate are usually used instead of chickpea flour due to their higher percentage of protein. Chickpea flour contains carbohydrate and lipids molecules which can reduce the rate of hydrolysis (62, 67).

**Figure 3:**
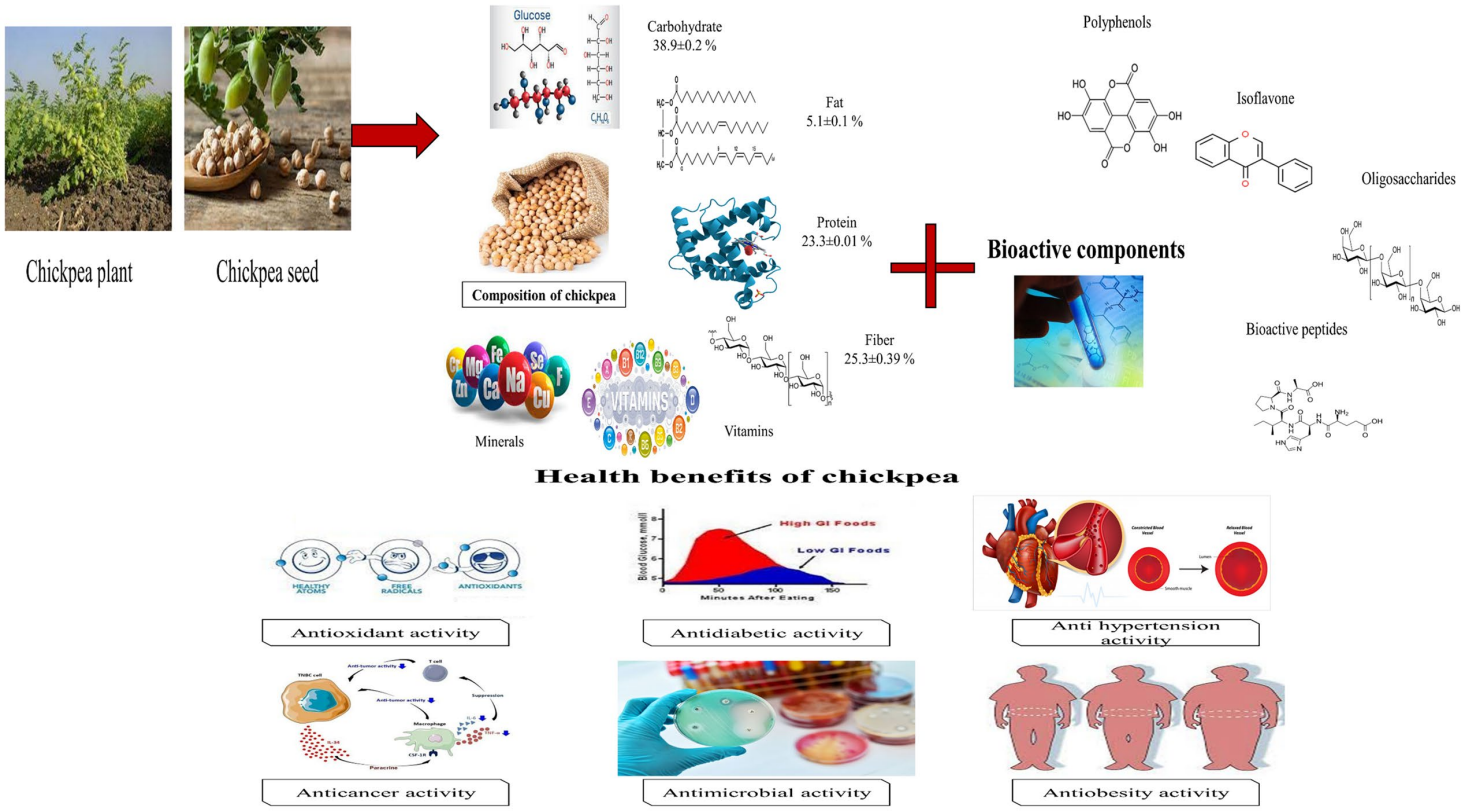
Schematic diagram of composition, bioactive components and health benefits of chickpea.

During the past 20 years various enzymes pepsin, papain, flavorzyme, alcalase, trypsin, and *α*-chymotrypsin used alone or in combination for the production of chickpea peptides. Further research is needed for the optimization of the production of chickpea peptides from chickpea (64). Antioxidant activity of beta-conglycinin and glycinin showed higher activity after enzyme hydrolysis, after enzyme hydrolysis more active R-group of amino acid will release and produce higher activity. The unfolding of hydrophobic amino acids also leads to increase the activity. The protein nature, degree of hydrolysis, and molecular mass of the released peptides are responsible to increase or decrease the hydrophobicity. A peptide having molecular mass < 1 kDa showed improved activity and bitter taste (63). The antioxidant activity of hydrolysate is effected by enzyme specificity, amino acid sequence, and composition of peptides, peptide size widely depends on the type of enzyme used for hydrolysis. In other research used soy protein isolate, various enzymes were used to produce hydrolysate with different degrees of hydrolysis. A mixture of the peptide was produced and showed antioxidant activity according to the degree of hydrolysis (68). Hydrolysis of chickpea protein by alcalase showed higher antioxidant activity. Reducing power and hydroxyl radical scavenging activity of chickpea protein hydrolysate improved by combined treatment with alcalase and ultrasonic treatment with a degree of hydrolysis of 20.03%. Besides this chromatographic fractionation was performed to isolate a novel peptide from alcalase chickpea protein hydrolysate which showed hemolytic activity on bovine erythrocytes (69). It is stated that aclalase-derived peptides have the low molecular weight and various functional properties as compared to other enzymes (70). Barbana and Boye (31) reported that sequential hydrolysis of chickpea protein with alcalase and flavorzyme generates higher DH (58.89–78.58%). It reveal that the combine use of Exo and endo-proteases improve the degree of hydrolysis and N-terminal sides, alternately improving the functional properties of hydrolysate (Figure 3).

## 4. Health benefits of chickpea peptide and hydrolysate

### 4.1. Antioxidant activity

Food quality and human health both are affected by free radicals, in body macromolecules such as carbohydrate nucleic acids (DNA and RNA), polymers, and unsaturated lipids are damaged by unstable free radicals, causing oxidative stress. As a result many health problems such as arteriosclerosis, diabetes mellitus, neurodegenerative diseases, cancer, inflammatory diseases, and arteriosclerosis (71). While in food oxidation occurs in the presence of free radicals, which cause a change in food texture, color and flavor, and nutritive quality (1).

Several synthetic antioxidants, i.e., butylated hydroxytoluene, butylated hydroxyanisole, and propyl gallate have been used in the food industry for food preservation. However, use of synthetic antioxidants causes possible health hazard and food stability problems. Natural antioxidants could be the best option to replace synthetic antioxidants. Several natural compounds such as vitamin E, phenolic acids, flavonoids, ascorbic acid, carotenoids, protein and protein hydrolysates, and peptides retain antioxidant activity. Different protein sources have been used to generate peptides and hydrolysates having antioxidant activity, it present admirable options to be used to prevent oxidative stress, and used this protein sources as a natural antioxidants and nutritional supplement (72).

To upgrade the functional and nutritional characteristics of proteins, enzymatic hydrolysis is widely used to produce antioxidant peptides. A study reported that low molecular mass peptides having hydrophobic amino acids such as Val or Leu in their N-terminal showed higher antioxidant activity. Stronger antioxidant activity was observed in the presence of Tyr, Phe, and Trp (aromatic amino acids) and Met and Cys (sulfur-containing amino acids) (73). Chickpea protein and hydrolysates by various enzymes have been reported by various researchers. By the use of different biochemical assays, antioxidant activity of hydrolysate and peptides have been reported. Hydrolysates and peptides obtained from chickpeas showing antioxidant activity are presented in Table 8.

**Table 8:** Chickpea peptides and hydrolysate having antioxidant properties.

Peptide source	Preparation method	Obtained peptides	*In vitro*, *in vivo* antioxidant property	References
Chickpea	Germination	Low molecular weight peptides (9.4–10.9 kDa)	DPPH	(74)
Chickpea protein	Pepsin, pancreatin, affinity chromatography with immobilized copper, SEC, HPLC	Low molecular weight peptides (105–1205 Da)	Cu2+ chelation	(32)
Chickpea sprout protein	Trypsin, Neutrase, Alcalase, papain, IEC, SEC, RP-HPLC	LTEIIP (685.41 Da)	DPPH, HRSA	(75)
Chickpea protein	Alcalase, SEC	NFYHE (717.37 Da)	DPPH, HRSA, SRSA, Cu2+ and Fe2+ chelation, inhibition of linoleic acid autoxidation	(8)
Chickpea seeds	Pepsin, pancreatin, UF	Low molecular weight peptides (3.5–7 kDa)	ABTS, DPPH, Fe2+ and Cu2+ chelation	(74)
Chickpea protein	Alcalase, SEC	Low molecular weight peptides (two peaks of 940–2622 Da and 220–940 Da)	Reducing power, DPPH, MRSA, SRSA, inhibition of linoleic acid autoxidation	(76)
Chickpea protein concentrate	Alcalase, SEC, RP-HPLC	DHG (327.33 Da), VGDI (402.49 Da)	DPPH, reducing power, inhibition of lipid oxidation, Fe2+ chelation	(65)
Chickpea protein isolates	RP-HPLC	ALEPDHR (836.2 Da), TETWNPNHPEL (1336.3 Da), FVPH (498.1 Da), SAEHGSLH (836.1 Da)	Reducing power, FRSA, inhibition of peroxyl-induced oxidation in Caco-2 cells	(77)
Chickpea seeds protein	Alcalase, Flavourzyme	Hydrolysates	DPPH, inhibition of lipid oxidation	(78)
Chickpea protein	Alcalase, Flavourzyme, SEC	RQSHFANAQP (1,155 Da)	DPPH, ABTS, HRSA	(21)
Chickpea protein (albumins or globulins)	Alcalase enzyme, UF, RP-UPLC-DAD	FEI, FEL, and FIE	ABTS, DPPH	(79)
Chickpea sprout protein	(Trypsin, neutrase, alcalase and papain) IEC, GFC, RP-HPLC, MALDI-TOF-MS/MS	LTEIIP (Leu-Thr-Glu-IIe-IIe-Pro) with 685.41 Da	DPPH, OH	(75)
Chickpea protein concentrate	Alcalase, GF, RP-HPLC, ESI-MS and ESIMS/MS	Asp-His-Gly and Val-Gly-Asp-Ile	DPPH	(35)
Chickpea protein hydrolysate	MAL-DI-TOF-TOF	Asn-Arg-Try-His-Glu (molecular weight, 717.37 Da)	Caco-2 cell line (*in vivo*), Catalase activity, Glutathione reductase (GR) activity, Glutathione peroxidase (GP) activity	(80)

Quintero-Soto et al. (79) studied the antioxidant activity of chickpea protein (albumin and globulin) produced by alcalase. Obtained peak showed the highest activity was purified for the peptide sequence, three similar structure peptides have purified (FEI, FEL, FIE). Peptide fraction AH1-5 was evaluated for ABTS and DPPH activity (90.2 mg/mL) and activity ranged from 37.39 to 78.25% for ABTS and 73.65–74.14% for DPPH. The obtained values were higher than those obtained by Zhang et al. (81) who found antioxidant activity for soy hydrolysate synthetic peptides (81). The reason behind the highest ABTS activity is the presence of aromatic amino acids, the benzene ring is responsible to stabilize the free radicals by the donation of the electron. The antioxidant activity is also higher due to the presence of leucine at the C-terminus (82). In the case of DPPH activity higher activity was found in peptides having the highest hydrophobicity.

A study carried out by another researcher hydrolyzed chickpea sprout protein by various enzymes (trypsin, nutrase, alcalase, and papain). They found the nutrase fraction has the highest DPPH and OH activity. The hydrolysate was purified by IEC, GFC, RP-HPLC, and MALDI-TOF-MS/MS. The fraction having the highest DPPH and OH activity was identified for the amino acid sequence, the molecular weight of the peptide was 685.41 Da and the amino acid sequence was LTEIIP (Thr-Glu-IIe-IIe-Pro). Results suggested that peptides obtained from plant proteins having antioxidant activity are commonly short-chain amino acids (less than 15 amino acids) and contain Leu, Pro, Val, and Ile (hydrophobic amino acids) and Asn and Asp, Gln, and Glu (acidic amino acids) (75). Enzymatic hydrolysis of chickpea protein concentrate used as starting material, two peptides P_3_ and P_8_ purified showing higher antioxidant activity. The molecular mass of these peptides were 327, 33, and 402, 49, and the amino acid sequence was Asp-His-Gly and Val-Gly-Asp-Ile. As compared to P_3_, P_8_ exhibit higher antioxidant activity. In another study, four antioxidant peptides (ALEPDHR, TETWNPNHPEL, FVPH, and SAEHGSLH) from chickpea protein hydrolysate were reported and their antioxidant activity was studied in CaCo 2 cells. Three peptides SAEHGSLH, FVPH, and TETWNPNHPEL showed higher cellular antioxidant activity while one peptide ALEPDHR showed lower activity (83).

### 4.2. Diabetes

Diabetes mellitus can be defined as a chronic disease which results in an increase in blood sugar levels as a result of the pancreas producing insufficient insulin (84). Plant-based diet is widely used in the management and prevention of diabetes (85). Chronic metabolic disease diabetes represents a globally health problem with strong health and socioeconomic impact. About 90% of the world’s diabetic population is suffering from type 2 diabetes mellitus, the number is growing rapidly reaching up to 439 million by 2030 (86). Type 2 diabetes is also associated with other chronic diseases such as retinal damage, neuropathy, chronic renal failure, microangiopathy, and cardiovascular disease. The cost of diabetes mellitus will reach up to 2.5 million USD in 2030. This indicates that there is an urgent need to control and prevent type 2 diabetes (87).

Synthetic medicines like voglibose and acarbose are widely used for the control of type 2 diabetes, which inhibit the carbohydrate hydrolyzing enzymes (α-glucosidase, α- amylase) and suppress glucose absorption. These drugs have some side effects such as flatulence (78% of patients) and diarrhea (14% of patients) and the costs of these drugs are also high (88). Another approach to controlling type 2 diabetes is the inhibition of the dipeptidyl peptidase-IV enzyme. Several inhibitors such as sitagliptin, saxagliptin, linagliptin, and vildagliptin which are collectively called gliptins, cannot control glycemic load adequately and also have some side effects such as headaches, weight gain, urinary and upper respiratory tract infections, hypoglycemia and cardiovascular problems (89). There is a complex mechanism in the body to regulate insulin production and control the glucose level. Currently, the search for natural therapeutic products which have limited or no side effects for the treatment of disease is growing. For the management of type 2 diabetes food-derived compounds such as flavonoids, phenols, proteins and peptides, which have the potential for an antidiabetic activity is of research interest. Recent research reported that some proteins, amino acids, peptides, and protein hydrolysates play important role in blood glucose regulation.

GI (glycemic index) can be defined as the ability of various foods to raise the blood glucose level. The glycemic conditions must be controlled by the persons having diabetes. People must choose foods with carbohydrate sources which cause a slow release of glucose after consumption. Foods considered a good source of energy and carbohydrates for diabetic persons are beans and pulses having lower GI, which help in the regulation of insulin secretion and glycemic condition in type 2 diabetic persons (90). Significant reduction in triglyceride and glucose levels was observed in diabetic rats by feeding chickpea seeds with a dose of (400 mg/kg). The study reported that bromelain-producing chickpea hydrolysate has a peptide sequences containing glycine amino acid. The length of amino acid chains ranged between 7 and 18 amino acids. The peptide contains hydrophobic amino acids having the potential to inhibit type 2 diabetes.

Chandrasekaran and De Mejia (91) investigated peptide sequences obtained from germinated chickpea isolated protein produced by ficin enzyme. Three peptide sequences SPGAGKG, GLAR, and STSA were isolated by LC-ESI-MS/MS. The antidiabetic potential of peptides was evaluated through DPP-IV and α-glucosidase inhibition. Results showed that SPGAGKG is more active than GLAR to inhibit both α-glucosidase and DPP-IV inhibition. Chickpea peptide can be used as a potential source for the production of functional food ingredients. Chickpea peptides and hydrolysate having antidiabetic properties are depicted in Table 9.

**Table 9:** Chickpea peptides and hydrolysate having antidiabetic properties.

Peptide source	Preparation method	Obtained peptides	*In vitro*, *in vivo* antidiabetic property	References
Chickpea protein hydrolysate	Bromelain enzyme, LC-ESI/MS	GKGSGAF	Inhibit DPP-IV	(84)
Chickpea protein hydrolysate	Bromelain enzyme, LC-ESI/MS	RASAAGGGGGGVSSR	Inhibit DPP-IV	
Chickpea protein hydrolysate	Bromelain enzyme, LC-ESI/MS	QNPLSSAAPTGAGKPY	Inhibit DPP-IV	
Chickpea protein hydrolysate	Bromelain enzyme, LC-ESI/MS	AMMELGWSTSGEFLL	Inhibit DPP-IV	
Chickpea protein hydrolysate	Bromelain enzyme, LC-ESI/MS	TRGTGGR	Inhibit DPP-IV	
Chickpea protein hydrolysate	Bromelain enzyme, LC-ESI/MS	LLGELCGSGNTVVEL	Inhibit alpha-glucosidase, DPP-IV, DPP-III	
Chickpea protein hydrolysate	Bromelain enzyme, LC-ESI/MS	GKAAPGSGGGTKA	alpha-glucosidase, DPP-IV, DPP-III	
Chickpea protein hydrolysate	Bromelain enzyme, LC-ESI/MS	GLTQGASLAGSGAPSPLF	Inhibit alpha-glucosidase, DPP-IV, DPP-III	
Chickpea protein hydrolysate	Bromelain enzyme, LC-ESI/MS	KSGGGGGGTAVT	DPP-IV, DPP-III	
Chickpea protein hydrolysate	Bromelain enzyme, LC-ESI/MS	NKKSGAGGGSGAGKGGVA	Inhibit DPP-IV	
Chickpea protein hydrolysate	Bromelain enzyme, LC-ESI/MS	KMTAGSGVT	Inhibit DPP-IV	
Germinated chickpea isolate protein	Papain or ficin enzyme (LC-ESI-MS/MS)	SPGAGKG, GLAR, and STSA	*In vivo* inhibition of DPP-IV and α-glucosidase	(91)
Chickpea protein hydrolysate	Pepsin-pancreatin, LC-MSMS	SPKGAGF	dipeptidyl peptidase IV (DPPIV) inhibition	(92)
Chickpea protein hydrolysate	Pepsin-pancreatin, LC-MSMS	NGPGPA	dipeptidyl peptidase IV (DPPIV) inhibition	
Chickpea protein hydrolysate	Pepsin-pancreatin, LC-MSMS	GKGSAT	dipeptidyl peptidase IV (DPPIV) inhibition	
Chickpea protein hydrolysate	Pepsin-pancreatin, LC-MSMS	SPKGAGF	dipeptidyl peptidase IV (DPPIV) inhibition	

### 4.3. Hypertension

Hypertension or high blood pressure is the leading risk factor for heart disease. Other diseases such as heart failure and renal disease, stroke, and myocardial infarction are closely related to hypertension. About 9.4 million people death occurs due to the aforementioned diseases. Cardiovascular function and blood pressure are regulated by dipeptidyl carboxypeptidase angiotensin I–converting enzyme (EC 3.4.15.1), which converts the inactive decapeptide angiotensin I into the potent vasoconstricting octapeptide angiotensin II, by removing dipeptide from the C-terminus, which can increase blood pressure. ACE inhibitors help to reduce the occurrence of hypertension, there is still confusion that which option is best either to inhibit the receptors that bind angiotensin II and signal vasoconstriction or ACE directly (93). Many drugs used for the treatment of blood pressure are related to the inhibition of angiotensin-I converting enzyme. Synthetic drugs (captopril, enalapril, and Lisinopril) are useful for a short time use, but these drugs have serious side effects. To overcome the disadvantages of synthetic drugs researchers are interested to find natural inhibitors for angiotensin I-converting enzymes. In this regards peptide have some inhibitory function for angiotensin I-converting enzyme gaining popularity (94).

Several peptides from food sources, i.e., legumes have the potential to inhibit angiotensin I-converting enzyme. Chickpea protein and hydrolysate showed ACE 50% activity is reported and the methods used for the study are well standardized. Chickpea hydrolysate produced by papain enzyme is the most potent sample for the inhibition of the ACE enzyme (IC50 = 0.010 μg/mL). ACE inhibitory activity of chickpea flour hydrolysate also showed activity (95). Previous research reported four ACE inhibitory peptides from enzymatic hydrolysate of chickpea protein having low molecular weight < 1 kDa, having ACE inhibitory activity IC50 0.1 mg/mL. Two of the peptides showed strong activity. Higher amino acid residue number of peptides shows the highest activity (96). In another study protein hydrolysate obtained from chickpea desi variety having molecular weight < 4 kDa exhibited strong ACE activity with the IC50 of 140 ± 1 μg/mL (31). To obtain the single chickpea peptide effect on ACE inhibition, studies are required to check the activity in animal models and clinical studies to confirm the discussed outcomes. Chickpea peptides and hydrolysate having antihypertensive properties are given in Table 10.

**Table 10:** Chickpea peptides and hydrolysate having antihypertensive properties.

Peptide source	Preparation method	Obtained peptides	*In vitro*, *in vivo* antihypertensive property (IC_50_ mg/ml)	References
Chickpea flour	Alcalase,gel filtration, FPLC, RP-HPLC	Met:Asp	0.021	(97)
Chickpea flour	Alcalase,gel filtration, FPLC, RP-HPLC	Met:Phe:Asp:Leu	0.021	
Chickpea flour	Alcalase,gel filtration, FPLC, RP-HPLC	Met:Asp:Phe:Leu:Ile	0.011	
Chickpea flour	Alcalase,gel filtration, FPLC, RP-HPLC	Met:Asp:Leu:Ala	0.013	
Chickpea flour	Alcalase,gel filtration, FPLC, RP-HPLC	Met:Asp:Leu	0.021	
Chickpea seed	Dry seeds		0.7	(98)
Chickpea protein	Alcalase, papain, pancreatin	Hydrolysate	0.696, 0.030, 16.340	(99)
Chickpea protein isolate	Alcalase, FPLC, gel filtration, RP-HPLC	Peptide molecular mass < 1 kDa	0.108, 0.117	(96)
Chickpea protein concentrate (Desi and Kabuli)	*in vitro* gastrointestinal simulation, alcalase/ flavourzyme, and papain, SDS–PAGE and SE-HPLC	Hyrolysate peptides Molecular mass < 4 kDa	0.229, 0.282, 0.316, 0.140, 0.180, 0.228	(31)

### 4.4. Cancer

Cancer is one of the leading causes of death, having high incidence worldwide, it is a chronic non-communicable disease described by invasion and metastasis, uncontrolled cell replication, increased proliferation, resistance to apoptosis, and evasion of tumor suppressor genes. 14.1 million cases of cancer were reported in 2012, while in 2030 it is estimated that 22 million new cases and 13.2 million deaths will occur (100). Initiation, promotion, and progression are the three stages of cancer genesis and evolution. Vascular invasion, survival of tumor cells, metastasis, and progression of malignancy are related to the overexpression of PCNA (101). Among all types of cancer colon cancer is the third most frequently occurring cancer type worldwide in both genders. Various factors (genetic and epigenetic) are related to its pathogenesis. Some case–control studies reveal the fact that hypercaloric diets rich in fats and carbohydrates are mainly responsible for the development of cancer (102). After hydrolyzing some foods especially protein generate some compounds (peptides) with various biological activities. Peptides obtained from soybean, beans, and chickpea protein hydrolysate having hypolipidemic, antioxidant, antiproliferative, metal chelating, an inhibitor of the angiotensin-converting enzyme, hypocholesterolemic and anticarcinogenic activities (100). Chickpea consumption is reported to have health benefits due to its composition of complex carbohydrates and protein. Real Hernandez et al. (64) reported two animal studies that reported the anticarcinogenic activity of hydrolysate of chickpea protein. Sequential hydrolysis of chickpea protein concentrate by pepsin followed by pancreatin did not significantly decrease the number of aberrant crypts in mice with cancer, but was helpful in the regulation of cholesterol homeostasis in mice. Chickpea albumin hydrolysate formed by alcalase followed by Flavourzyme fed to mice (100 mg per kilogram of body weight/day) has significantly inhibited and reduced the number of tumors in mic. However human studies are still required to clear that chickpea protein hydrolysates and peptides exhibit anticarcinogenic effects. The previously mentioned peptide (RQSHFANAQP) having hypolipidemic and antioxidant activity, was also reported to decrease the capability of cancer cells. Peptide (RQSHFANAQP) fragment ANAQ is also found to interact with protein p53 *in silico*, which is actively involved in the cancer progression. However to confirm to the role of chickpea peptide and hydrolysate in cancer prevention more interventional research is required.

### 4.5. Other health benefits

Antidiabetic, anticancer, antihypertensive, and antioxidant are not only the bioactivities of chickpea peptides that have been reported. Peptides and hydrolysate of chickpeas possess some other activities such as antimicrobial, anti-inflammatory, hypocholesterolemic, and some skin health-promoting activities. Pathogenic and spoilage bacteria spoil the food and decrease the shelf life as well damage the food safety in the food industry. The food industry tried natural and synthetic antibacterial agents to avoid such damage occurred by bacteria. Due to the negative effect of the chemicals on human health, the food industry tried to restrict these antibacterial agents (103). To avoid toxic effects there is a need to search natural biomolecules. In this case, bioactive peptides can be used as alternatives to the synthetic antibiotics in food and pharmaceutical industries due to their low toxicity and high specificity (104). Such bioactive peptides can be obtained from food protein sources such as vertebrates, fish, eggs, wheat, insects, plants, milk, and bacteria (105). In the last year some plant proteins have been identified to prevent the development of microorganisms (106). Chickpea peptides were isolated some years ago, these peptides were arietin (5.6 kDa) and cicerin (8.2 kDa), and having antifungal activity against *Botrytis cinerea*, *Mycosphaerella arachidicola*, and *Fusarium oxysporum*. Another study also reported that peptide having molecular mass 8 kDa (Val-Lys-Ser-Thr-Gly-Arg-Ala-Asp-Asp-Asp-Leu-Ala-Val-Lys-Thr-Lys-Tyr-Leu-Pro-Pro) purified from green chickpea showed activity against *Mycosphaerella arachidicola*, *B. cinerea* and Physalospora piricola (107).

A physiological process that starts in the response to biological, chemical, or physical injury is called inflammation. This response is the protection of the body against infection, some chronic diseases such as cancer obesity, rheumatoid arthritis, diabetes, osteoporosis, and cardiovascular diseases, occur if the inflammation becomes chronic and excessive production of mediators such as tumor necrosis factor (TNF-α) and interleukin (1 and 6) are the major cause. Several drugs are used for the treatment of these diseases, but drugs have some side effects. To control the production of these mediators and to overcome the side effects of the drugs, researchers are trying to search for alternative treatments from natural sources (108). Chickpea seeds have been found to have some molecules possess the ability to inhibit the process of inflammation through various mechanisms. Recently study reported that chickpea extract has the potential to reduce the activity of cyclooxygenase-2 (COX-2), nuclear factor kappa B (NF-ĸB), and TNF-α in rats (109). Another study reveal that peptides fraction and phenolic extract at the concentration of (5 mg/mL and 0.5 mg/mL) have the ability to inhibit the production of nitric oxide in RAW 264.7 macrophages induced by lipopolysaccharides (LPS) (110). Peptide (RQSHFANAQP) purified from chickpeas have significantly reduced the expression of tumor necrosis factor- α in rats that fed on 20 mg peptide/kg of body weight/day (64). Another study reported that germinated chickpea protein hydrolysate contains hydrophobic peptides which inhibit the production of NO (nitric oxide) an inflammatory signaling molecule, but the sequence of the peptide was unknown (110). The peptide (LHQNIGSSSSPDIYNPQAGR) significantly reduced human *α*-amylase, but these findings also need some further verification (111). Intensive research is required on the anti-inflammatory effect of peptide and hydrolysate obtained from chickpea.

The high consumption of saturated fat, low consumption of dietary fiber, and low physical activity, cause high concentrations of cholesterol and lipid in the blood. Various metabolic and cardiovascular disorders such as coronary cardiopathy, atherosclerosis, obesity, pancreatitis, and fatty liver are linked with hyperlipidemia. To reduce the blood lipid level in the blood researchers are interested to search for bioactive compounds which can be used for this purpose. Recent research reported that the consumption of chickpea has been associated with a reduction of blood lipid level due to their high fiber and low lipid composition (112). Hydrolysate obtained from chickpea albumin by alcalase-flavourzyme was fed (150 mg/kg) to mice with a high-fat diet. The result showed an increase in high-density lipoprotein (HDL) cholesterol levels by 46.75, 36.55, 48.53, and 15.34%, respectively, and a decrease in total triglycerides in serum, LDL cholesterol, and cholesterol levels (113). Another study reveal a 50% reduction in the cholesterol level by the consumption of chickpea hydrolysate obtained through flavourzyme and alcalase (96). A peptide having amino acid sequence Arg-Gln-Ser-His-Phe-Ala-Asn-Ala-Gln-Pro from chickpea protein demonstrated to Kunming rats with a high-fat diet for 4 weeks, results attained decrease in serum and hepatic triglycerides and cholesterol level (114). Hydrolysate obtained through pepsin-pancreatin enzyme from chickpea protein showed a reduction in the concentration of LDL, of triglycerides level in mice. This effect is due to the concentration of hydrophobic amino acids present in hydrolysate, which are helpful in the excretion of lipids in feces (100). Significantly increased HDL level and reduction in triglycerides, total cholesterol, and LDL contents in serum were observed in obese rats fed with a fat-rich diet with chickpea peptide (112).

## 5. Conclusion

This review summarized information about the nutritional benefits of chickpea and its role in the health improvement. Chickpea consumption not only provide basic nutrition but also have health benefits. It is a cheap and easily available source of health-promoting fatty acids, carbohydrates, minerals and vitamins, folate, protein and b-carotene. Chickpea provide potential health benefits and lowering the development and progression of numerous chronic diseases (CVD, type-2 diabetes, etc.). Chickpea also contain bioactive peptides, which can produced through enzymatic hydrolysis. Peptide can be produced with food approved enzymes such as alcalase, flavorzyme, chymotrypsin, pepsin, papain, trypsin and pancreatin. Chickpea hydrolysate and peptides also have been health outcomes such as anticarcinogenic, skin health promoting bioactivities, anti-inflammatory, hypolipidemic, antioxidant, antihypertensive, antifungal, and antidiabetic. Some peptides such as RQSHFANAQP, NRYHE and VFVRN purified from chickpea showed health benefits but human studies are still required to conform the biological activity of these peptides in humans. Chickpea peptide and hydrolysate and peptides can be produced enzymatically and added to food products to promote human health. However research should be carried out to provide evidence and unravel the mechanism of chickpea peptide and hydrolysate involved in the disease prevention and promoting human health. Processing methods required for the production of bioactive peptides and to reduce the production of harmful (antinutrients compounds) need attention.

## Author contributions

NB and QK contributed to write the first draft of the manuscript, conception, and design of the study. IH organized the database and performed the statistical analysis. DL revised and supervised the work. All authors contributed to manuscript revision, read, and approved the submitted version.
